# Performance of Stratification Scores on the Risk of Stroke After a Transient Ischemic Attack: A Systematic Review and Network Meta-Analysis

**DOI:** 10.3390/jcm14176268

**Published:** 2025-09-05

**Authors:** Dimitrios Deris, Sabrina Mastroianni, Jonathan Kan, Areti Angeliki Veroniki, Mukul Sharma, Raed A. Joundi, Ashkan Shoamanesh, Abhilekh Srivastava, Aristeidis H. Katsanos

**Affiliations:** 1 Department of Health Sciences, McMaster University, Hamilton, ON L8S 4L8, Canada; derisd@mcmaster.ca (D.D.); sabrina.mastroianni@medportal.ca (S.M.); 2 School of Interdisciplinary Science, McMaster University, Hamilton, ON L8S 4L8, Canada; kanj13@mcmaster.ca; 3 Li Ka Shing Knowledge Institute, Toronto, ON M5B 1W8, Canada; argie.veroniki@gmail.com; 4 St. Michael’s Hospital, Toronto, ON M5B 1W8, Canada; 5 Department of Medicine (Neurology), McMaster University, Hamilton, ON L8S 4L8, Canada; mike.sharma@phri.ca (M.S.); raed.joundi@phri.ca (R.A.J.); ashkan.shoamanesh@phri.ca (A.S.); abhilekh.srivastava@medportal.ca (A.S.)

**Keywords:** transient ischemic attack, stroke prediction, clinical risk scores, risk stratification, diagnostic accuracy

## Abstract

**Background:** Patients after a transient ischemic attack (TIA) are at high risk of subsequent stroke. There are various scores that aim to accurately identify patients at the highest risk of stroke. However, without comparisons between these scores, it is still unknown which is the score with the best predictive utility. Our study aims to identify the risk stratification score with the highest utility to identify patients at high risk for stroke within 90 days after a TIA. **Methods**: The MEDLINE and Scopus databases were systematically searched on 1 December 2023 for observational cohort studies assessing the ability of a score to predict a stroke within the first 90 days from the index TIA event. Only studies that had a direct comparison of at least two scores were included. A random-effects network meta-analysis was performed. Sensitivity and specificity, along with relevant 95% credible intervals, and between-score and between-study heterogeneity were estimated. We also estimated relative sensitivities and relative specificities compared with the ABCD2 score. We ranked each score according to its predictive accuracy based on both sensitivity and specificity estimates, using the diagnostic odds ratio (DOR) and the summary receiver operating characteristic (SROC) curve. **Results:** Our systematic review highlighted 9 studies including 14 discrete cohorts. The performance of all scores to identify patients at high risk for stroke recurrence within 90 days following a TIA was low (pooled sensitivity range 48–64%, pooled specificity range 59–72%). In the network meta-analysis, we analyzed 6 studies with 11 discrete cohorts, including data from 8217 patients. The ABCD3-I score demonstrated the highest DOR, followed by the ESRS, ABCD, California, and ABCD2. The SROC curves demonstrate no significant differences in the performance of the scores, using the ABCD score as the common comparator. **Conclusions**: In this systematic review and network meta-analysis of observational cohort studies of patients who experienced TIA and were followed for the occurrence of subsequent stroke, we failed to identify a score performing significantly better for the prediction of stroke at 90 days. New models are needed for the prediction and stroke risk stratification following a TIA.

## 1. Introduction

Patients who experience a transient ischemic attack (TIA) are at a high risk of ischemic stroke, with a subsequent risk between 4 and 20% at 90 days post-TIA [1]. Accurately identifying patients who are at the highest risk of stroke after TIA is the first essential step in managing these patients and deciding who should be admitted into the hospital, as resource limitations do not allow for all patients presenting with a TIA to be admitted. Currently, there are numerous scores available to stratify patients at the highest risk of stroke after TIA, with each score having different criteria for what defines patients as high-risk, requiring emergent intervention [2].

Many studies have looked into various scores and clinical measures to stratify the risk for subsequent stroke following TIA. Some of the most clinically used scores include ABCD, ABCD2, and ABCD3-I. The ABCD score predicts 7-day risk of stroke by incorporating age (A), blood pressure (B), clinical features (C), and duration (D) of TIA [3], where a high-risk score is defined as ≥4. The ABCD2 score is a modified version of the ABCD score to incorporate diabetes while maintaining the same high-risk value of ≥4. It was originally developed to aid non-specialists in a community setting in the management of TIA patients [4]. Furthermore, the ABCD3-I score was developed after the ABCD2 to be used in a secondary care setting and includes information from initial diagnostic investigations [4]. The 3 added components are multiple TIA (≥2 TIA symptoms within 7 days), imaging criteria of carotid stenosis, and positive diffusion weighted imaging (DWI) [5], and a high-risk value was defined as ≥7 [4]. Other TIA risk stratification scores that have been used in the literature include the California score, ESRS (Essen Stroke Risk Score), and SPI-II (Stroke Prognosis Instrument II). The high-risk cut-offs are ≥2, ≥3, and ≥6, respectively. The California score was designed to help predict occurrence of stroke within 90 days of a TIA. It is less relevant for the immediate evaluation of stroke risk [6]. The ESRS is graded on a 10-point scale. It is used to predict the 1-year risk of recurrent stroke as well as combined cardiovascular events [6]. The categories included are hypertension, diabetes mellitus, smoking, prior stroke or TIA, prior myocardial infarction, stroke, peripheral artery disease, other cardiovascular disease, and congestive heart failure. The SPI-II is a modified clinical risk score of the SPI-I designed to estimate risk of recurrent stroke within 90 days of a TIA. The variables included are congestive heart failure, diabetes, prior stroke, age >70 years, stroke versus TIA, severe hypertension, and coronary artery disease [7].

Although each of these scores has utility for risk stratification, they also each have limitations. The ABCD2 score is currently the most widely used risk stratification tool, but it excludes symptoms that may suggest posterior circulation events, such as ataxia or dysmetria, and it does not account for TIA mechanisms and variables like ipsilateral large artery stenosis on imaging, intracranial atherosclerosis, carotid web, or atrial fibrillation—all of which may increase the risk of stroke after TIA [8]. Furthermore, while cerebral vascular imaging is important for guiding immediate management, many resource-limited settings do not have access to imaging modalities such as MRI and CT, so reliance on non-imaging variables becomes more important [9]. Resource limitations have made it difficult to implement multiple scores against one another in a similar clinical context [9].

Despite research comparing the individual scores as predictive measures for subsequent stroke risk following a TIA, an overall comparison of those scores is lacking. The purpose of this study was to determine which risk stratification score has the highest utility to identify patients at high risk for stroke recurrence within 90 days following a TIA.

## 2. Methods

This study is reported according to the PRISMA-NMA guidelines and followed methodological guidance from the Cochrane Handbook [10,11].

### 2.1. Data Sources and Search Strategy

A search was conducted between September 2023 and December 2023 for any studies prior to 2023 that derived or validated scores for the prediction of stroke after TIA or minor stroke on the databases MEDLINE and Scopus. The protocol of the study was registered in PROSPERO (CRD42022309046).

Reference lists of eligible studies and relevant systematic reviews were scanned manually for relevant studies. The following keywords were used for all searches: “TIA”, “transient ischemic attack”, “stroke”, “CVA”, “cerebral vascular accident”, “score”, “prediction”, and “risk”.

### 2.2. Study Selection

Observational cohort studies (prospective or retrospective) were included. Only studies reporting a direct comparison of at least two scores were included. Studies were considered for inclusion based on the relevance of data and information on the population assessed. Case series, case reports, and studies not published in English were excluded. If two or more studies with overlapping populations were found, the publication with the highest number of participants was chosen, and the other studies were excluded.

We included studies reporting a 2 × 2 table (number of true positives [TP], false negatives [FN], true negatives [TN], and false positives [FP]) or joint classification tables of scores predicting the risk of stroke. Studies that provided numerical estimates (e.g., sensitivity, specificity values) that allowed for the manual generation of a 2 × 2 table or joint classification tables were also included. Sensitivity corresponds to the probability that a score is positive in individuals with incident stroke during follow-up, and specificity refers to the probability that a score is negative in individuals without incident stroke during follow-up. Scores were estimated for their predictive accuracy and evaluated from their sensitivity and specificity estimates.

### 2.3. Outcome Measure

The main outcome of this study was the ability of a score to predict a stroke within the first 90 days from the index TIA event. We used sensitivity and specificity of each given score as effect measures to predict the risk of incident stroke within the first 90 days from the index TIA event. We also assessed the positive and negative predictive values of each score.

### 2.4. Within-Study Bias Assessment

The Quality in Prognostic Studies (QUIPS) tool [12] was applied to assess risk of bias in eligible prognostic factor studies. The six domains with associated signaling questions were used to guide the assessment of bias within each study. These six domains include study participation, study attrition, prognostic factor measurement, outcome measurement, study confounding, statistical analysis, and reporting [12]. The assessment was conducted by two researchers (SM, DD) independently, and there were no discrepancies between their ratings for each study.

### 2.5. Data Extraction and Quality Assessment

Data extraction was performed by two researchers (AS, AHK) independently through the use of an a priori standardized data extraction form. The extraction was performed according to the Checklist for Critical Appraisal and Data extraction for Systematic Review of Prediction Modelling Studies (CHARMS) [13]. From eligible studies, the 2 × 2 table was abstracted (TP, FN, TN, FP), year of publication, study type (prospective, retrospective, cohort), setting (single center, multicenter, hospital, ER-based, clinic-based), country, and participant characteristics (age, sex, vascular comorbidities, and antithrombotic treatments). Risk score characteristics such as cut-off values, time of test performance from TIA event, investigator performing the test, etc., were also abstracted. In order to determine the sensitivity and specificity for each score, the 2 × 2 table from each study was used. Each score had an associated high-risk cut-off, and the actual status was based on subsequent stroke after TIA (based on the time points indicated in each study).

### 2.6. Quantitative Synthesis

We modelled TP, TN, FP, and FN at all available cut-off values using the restricted maximum likelihood estimation from the inverse variance weighted linear mixed effect model with common random intercept and common slope [14]. The diagmeta R package (version 4.5.1, Vienna, Austria) [15] was used to run the meta-analysis. The model was informed by all TP, TN, FP, and FN values from multiple cut-offs reported in each study to derive the optimal cut-point of each score (maximum value of the weighted sum of sensitivity and specificity), separately, accounting for the between-study heterogeneity and correlation between scores. A summary receiver operating characteristic (SROC) curve and the area under the receiver operating characteristics (AUC) were used to pool the data from all the sources in each meta-analysis. The overall test accuracy is assessed by the closeness of the curve to the top left corner, with a hypothetical perfect test having an area under the curve equal to one, and a curve with a straight line having a positive test result half the time. A curve closer to the top left corner indicates higher sensitivity and a lower false positive rate (1−specificity). SROC curves were presented with relevant confidence regions. Summary sensitivity and specificity with associated 95% confidence intervals of each predictive score were reported.

A random-effects network meta-analysis was performed according to the model suggested by Nyaga et al. [16], accounting for correlations between scores within the same study. Their code was used to run the network meta-analysis within a Bayesian framework through the *rstan* package in R. Sensitivity and specificity, along with relevant 95% credible intervals, and between-score and between-study heterogeneity were estimated. We also estimated relative sensitivities and relative specificities compared with the ABCD2 score. Transitivity between test comparisons in the network was evaluated using a priori defined potential effect modifiers: age and prevalence of vascular risk factors. Transitivity relies on the assumption that clinical and methodological effect modifiers are generally comparable across test comparisons and that any missing data are missing at random. Intransitivity can create statistical inconsistency between direct and indirect evidence on the same test comparisons. Despite multiple methods that have been proposed to assess consistency in NMA of interventions, these methods are not yet available in diagnostic test accuracy-NMA [17,18,19]; thus, we did not evaluate the presence of inconsistency in the network. We ranked each score according to its predictive accuracy based on both sensitivity and specificity estimates, using the diagnostic odds ratio [20].

## 3. Results

**Figure 1:**
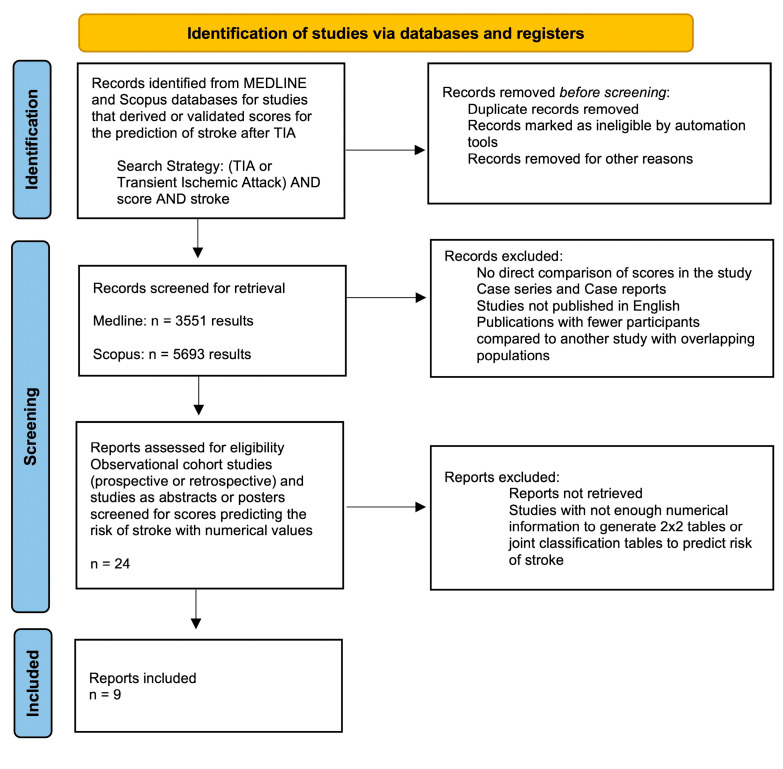
Results of study selection process.

The systematic database search yielded a total of 3551 and 5693 records from the MEDLINE and SCOPUS databases, respectively. After excluding duplicates and initial screening, we retrieved the full text of 24 records that were considered potentially eligible for inclusion. After reading the full-text articles, 15 were further excluded. Finally, we identified 9 eligible studies that qualified for inclusion in the systematic review and meta-analysis (Figure 1).

### 3.1. Quality Assessment

Using the QUIPS tool, the risk of bias of each of the studies included was assessed, and none of them were deemed to have overall high risk of bias (Table 1).

Overview of the population characteristics included in the 14 discrete cohorts of the 9 studies identified by the literature search is presented in Table 2.

### 3.2. Network Meta-Analysis

**Figure 2:**
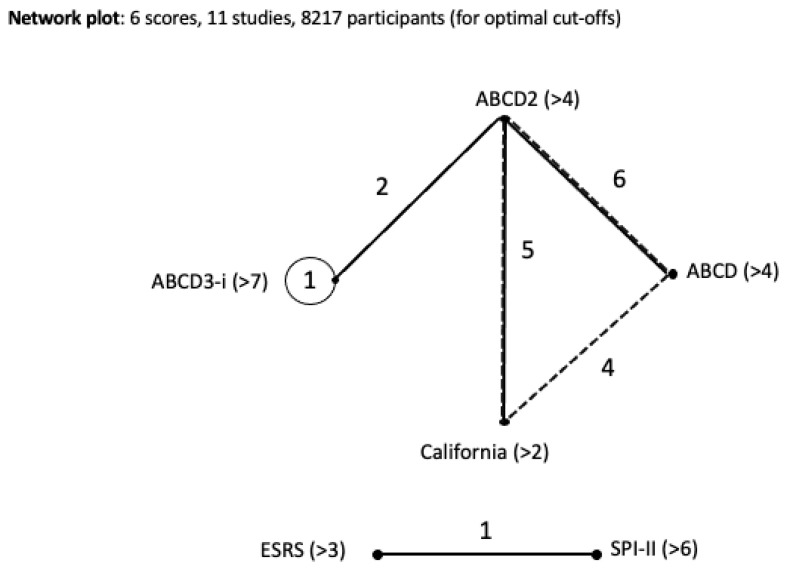
Network geometry of the studies included in the network meta-analysis. Each node represents a score at its optimal cut-off value, while each edge indicates at least one study comparing the connected scores. Closed circles denote scores assessed in single-score studies, with the numbers inside representing the number of such studies. Solid black dots (non-closed circles without an enclosed number) indicate scores evaluated only in paired- or multi-score studies. Dashed lines correspond to paired-score studies, while solid lines represent multiple-score studies.

We conducted a network meta-analysis of 6 studies with 11 discrete cohorts, excluding Dai et al. [21], De Marchis et al. [22], and Kiyohara et al. [23] as they did not provide information on the performance of their respective scores at optimal threshold. Our NMA included 8217 participants, and six scores at their optimal thresholds, as defined by the mixed effects model for each score separately; these included: ABCD2 (>4), ABCD (>4), ABCD3-I (>7), California (>2), ESRS (>3), SPI-II (>6). A network plot was derived to visualize the connectivity and geometry of the included studies [29] and is presented in Figure 2. The ABCD2 (>4) score was informed by the largest number of studies (*n* = 8), followed by the ABCD (>4) (*n* = 5) and California (>2) (*n* = 5) scores, and then the ABCD3-I (>7) (*n* = 3) score. The ESRS (>3) and SPI-II (>6) scores included in this meta-analysis did not have enough data to be connected to the remaining network and were both compared in one study. Most studies (*n* = 6) compared ABCD (>4) vs. ABCD2 (>4) scores, followed by the ABCD2 (>4) vs. California (>2) scores (*n* = 5), the California (>2) vs. ABCD (>4) scores (*n* = 4), the ABCD2 (>4) vs. ABCD3-I (>7) scores (*n* = 2), and the ESRS (>3) vs. SPI-II (>6) scores (*n* = 1).

Based on the demographics and vascular risk factors of the participants in each cohort (Supplemental Table S1), there was no evidence to suggest a violation of the transitivity assumption. Our network meta-analysis suggested that scores had comparable sensitivities and specificities at their optimal thresholds (Table 3). Scores with sensitivities below 65% indicate limited predictive value for clinical decision-making. Furthermore, it has been proposed that for a test to be considered clinically useful, the sum of its sensitivity and specificity should exceed 1.5—midway between 1 (a non-informative test) and 2 (a perfectly accurate test) [30].

Among scores, the ABCD3-I demonstrated the highest diagnostic odds ratio (DOR). This was followed by the ESRS, ABCD, California, and ABCD2. SPI-II showed the lowest DOR. Between-study heterogeneity, reported as standard deviations for sensitivity and specificity, varied across scores, with ABCD showing the least heterogeneity for sensitivity (SD = 0.16) and California showing the lowest for specificity (SD = 0.43). This data is portrayed in Table 4.

**Figure 3:**
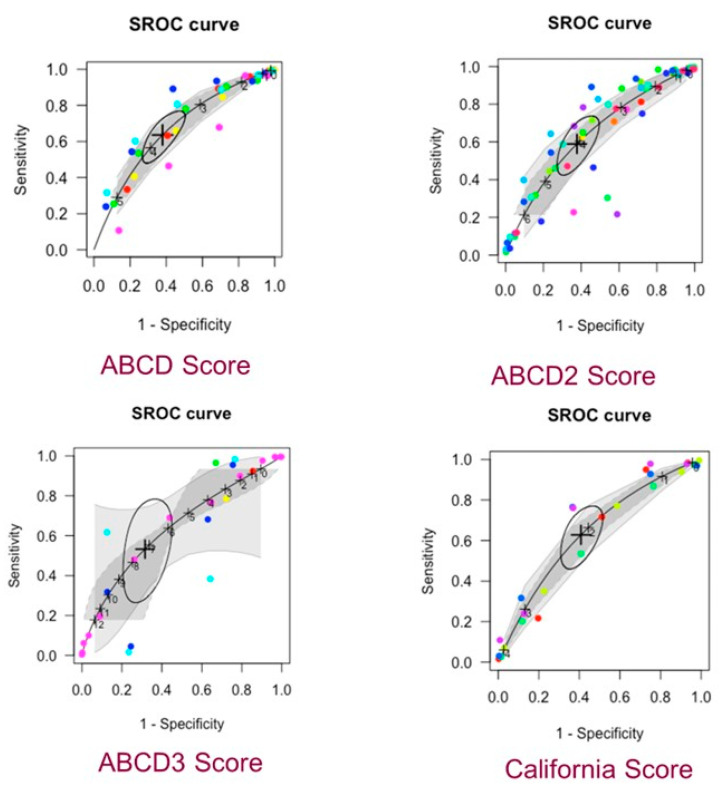
SROC curves for the main scores incorporated into this meta-analysis.

The SROC curves demonstrate no significant differences in the performance of the scores, using the ABCD score as the common comparator. None of the scores performed particularly well in accurately predicting the risk of subsequent stroke in patients following a TIA (Figure 3).

## 4. Discussion

We found that existing scores inadequately predict the risk of incident stroke within the first 90 days from a TIA event. The sensitivity of each score fell between the range of 48–64% and the specificity fell between the range of 59–72%. This renders the scores comparable as their ranges are similar. We also found no significant differences in the performance of the scores in accurately predicting the risk of stroke in patients following a TIA.

Based on the current literature, the poor predictability of stroke in TIA patients within the first 90 days is somewhat expected. While some scores have shown some usefulness, they have been inconsistent in their overall ability to predict stroke after a high-risk TIA across populations and clinical settings. A study conducted by Fothergil et al. [31] highlighted that relying on the ABCD2 misses some patients who have strokes within 7 days of a TIA. Furthermore, more studies have shown that the predictive value of the score is limited when not taking into account factors such as carotid stenosis and radiographic evidence of infarction [3,32]. Engelter et al. [26] refined the ABCD2 score to include the variables etiology and DWI-positivity, which was found to enhance the predictability of stroke after TIA. However, the meta-analysis by Zhao et al. [33] compared these scores, finding that both had relatively unsatisfactory predictive accuracy. Although the addition of imaging modalities like DWI-positivity may seem like an effective way to refine current TIA scores to risk-stratify patients, resource limitations may render such modifications less useful than intended in practice. With many patients being evaluated in community hospitals or primary practice settings when presenting with TIA symptoms, initial risk stratification based on more complex imaging modalities may not be practical in order to create a generalizable, useful score. A statement from the American Heart Association highlights these considerations, stating that TIA risk stratification scores do help identify high-risk patients and guide management, but given their limitations, TIA stratification scores should be part of a more comprehensive evaluation [8].

Current scores also fail to take into account demographic data that may help risk stratification of patients with a TIA. While risk factors such as age, history of hypertension, and diabetes mellitus are usually considered, some factors, such as tobacco smoking, are not taken into account. Also, there is a lack of data across populations, as well as differences between studies conducted in certain populations. The majority of the data used in our study was from Caucasian and Asian populations. Previous literature has shown examples of the variance between these populations, stating that Chinese populations have reported a higher incidence of stroke compared to European populations [34]. Most of the findings were significant for hemorrhagic stroke and did not have clear evidence for ischemic stroke subtypes. An American study by Trimble et al. [35] found that African Americans, Hispanics, and Native Americans all had higher stroke risks, stroke occurrences, and possibly more severe strokes when compared to non-Hispanic white people. These disparities highlight the need to incorporate demographic variables into future TIA scores. Acknowledging and addressing these gaps can lead to better risk stratification of patients and better outcomes post TIA.

There is a variety of other clinical limitations surrounding current TIA scores, as well as methodological challenges that present when attempting to evaluate their predictiveness. One significant limitation of current score systems is highlighted by their reliance on a limited set of variables. The study by Johnston et al. [28] discusses how some findings have shown the presence of weakness, older age, and diabetes as positive predictors of stroke after TIA. Amin et al. [8] argue that a TIA evaluation without MRI can potentially miss higher-risk patients who may require closer monitoring. Additionally, they discuss how factors such as laboratory and cardiac assessment should be considered. The absence of imaging-based predictors, specifically MRI findings, in many existing scores may reduce their capability to perform well. This is more apparent in high-risk subgroups, especially in patients presenting with atypical symptoms. In short, these studies suggest that future TIA scores should integrate more comprehensive clinical assessments as they aim to improve their predictive power. There are also important methodological limitations related to the heterogeneity of included populations. Lack of cross-cultural data can raise demographic concerns with the generalizability of our study’s findings. Specifically, this is an issue with underrepresentation of African American populations and other racialized groups in creation and validation of many TIA scores. This further contributes to a lack of generalizability of findings and differences in stroke risk profiles. Furthermore, effect modifiers such as study quality, participant age, and prevalence of risk factors were not explored in a sensitivity or subgroup analysis. There is also variability in secondary stroke prevention strategies across the different studies, as well as lack of central adjudication of stroke events amongst different centers.

There are also limitations of the scores surrounding the number of variables and factors they take into consideration. The majority of the scores mentioned use about 4–5 different factors to evaluate stroke risk. This calls into question the number of factors that should be considered when forming a reliable score. The Canadian TIA score is a score that utilizes up to 13 different variables. Additionally, it splits these variables up into both clinical findings as well as investigations conducted in the emergency department. The Canadian TIA score was validated in 2 large multicenter cohort studies and outperformed the ABCD2 in a study published by Perry et al. [36]. The study’s primary outcome was subsequent stroke within 7 days of a TIA, and it was, therefore, not included in our study. Despite the lack of data for a 90-day time frame, it is still worth noting that an increased number of variables can lead to improved outcomes. Furthermore, the division of variables accounts for the various settings where patients with TIA symptoms may present, including primary care settings or the ED. The exclusion of scores that do not fit within a 90-day time frame presents limitations in evaluating immediate/short-term risk assessment. This potentially biases our findings against scoring systems that perform well within shorter time frames.

## 5. Conclusions

Overall, our meta-analysis highlights that while existing TIA scores provide some level of risk stratification, they fall short in accurately predicting stroke incidence within 90 days after a TIA. While some scores do show better predictability relative to others, there is no significant data that favors one over the other, and all scores remain suboptimal. This study shows the need for change in the way TIA scores are developed and utilized. Future tools may benefit from imaging variables such as the inclusion of DWI sequence information from MRI, which has demonstrated predictive value beyond clinical scoring systems [37]. Furthermore, there is room for the integration of future technology, such as machine learning, in combining high-risk clinical features and imaging qualities to deliver personalized risk assessments for patients [38,39]. Developing robust scoring systems that incorporate a variety of predictors is imperative to the development of new scores that can reliably stratify the risk of stroke following a TIA. Those scores should be externally validated in diverse populations to ensure their consistency and generalizability.

## Figures and Tables

**Table 1:** QUIPS Assessment for studies included.

Study	QUIPS Issues to Consider						
	Study Participation	Study Attrition	Prognostic Factor Measurement	Outcome Measurement	Study Confounding	Statistical Analysis and Reporting	Overall
Dai et al., 2015 [21]	Low	Low	Moderate	Low	High	Low	Low-moderate
De Marchis et al., 2014 [22]	Moderate	Low	Low	Low	High	Low	Low-moderate
Kiyohara et al., 2014 [23]	Low	Low	Moderate	Moderate	Low	Low	Low-moderate
Knoflach et al., 2016 [24]	Low	Moderate	Low	Low	Moderate	Low	Low-moderate
Song et al., 2013 [25]	Low	Moderate	Low	Low	Low	Low	Low
Ildstad et al., 2021 [4]	Low	High	Moderate	Low	Moderate	Low	Low-moderate
Engelter et al., 2011 [26]	Low	Low	Low	Low	Moderate	Low	Low
Liu et al., 2013 [27]	Moderate	High	Moderate	Low	High	Low	Moderate
Johnston et al., 2007 [28]	Low	High	Low	Low	Moderate	Low	Low-moderate

**Table 2:** Characteristics of studies included in the systematic review.

Cohort Study	Location	Sample Source	Scores Compared	Type of Study	Participants	Timeline	Mean Age	% Female
Dai et al., 2015 [21]	Nanjing, China	Nanjing Stroke Registry Program	ABCD2 ABCD3-I	Registry	658	July 2009–December 2013	62	26.7
De Marchis et al., 2014 [22]	Switzerland & Germany	University Hospitals ED	ABCD2 ABCD3-I Copeptin value	Cohort	302	March 2009–April 2011	69	37.1
Kiyohara et al., 2014 [23]	Fukuoka, Japan	Fukuoka Stroke Registry	ABCD2 ABCD3 ABCD3-I	Registry	693	June 2007–August 2023	69	37.8
Knoflach et al., 2016 [24]	Austria	Austrian Stroke Unit Registry	ABCD2 ABCD3-I	Registry	2457	December 2010–January 2014	71.9	34.3
Song et al., 2013 [25]	Zhengzhou, China	Zhengzho University-affiliated hospital database	ABCD ABCD-I	Cohort	239	October 2010–December 2011	57.4	40.2
Ildstad et al., 2021 [4]	Norway	8 Central Norway region hospitals	ABCD2 ABCD3-I	Cohort	305	October 2012–July 2014	68	40
Engelter et al., 2011 [26]	Switzerland	Basel Stroke Unit program registry	ABCD ABCDE+	Registry	248	November 2006–November 2008	70	40
Liu et al., 2013 [27]	Shikiazhuang, China	The Third Hospital of Hebei Medical University and the CHANCE database	ESRS SPI-II	Cohort	167	March 2009–October 2011	61.1	28.7
Johnston et al., 2007 [28]	San Francisco, California (USA)	16 California Emergency Departments	California and ABCD vs. ABCD2	Cohort	1707	May 1997–February 1998	Not reported	53
Johnston et al., 2007 [28]	Oxfordshire, UK	10 Family practices	California and ABCD vs. ABCD2	Cohort	203	January 1981–December 1986	Not reported	46
Johnston et al., 2007 [28]	San Francisco, California (USA)	16 California Emergency Departments	California and ABCD vs. ABCD2	Cohort	1069	March 1998–February 1999	Not reported	52
Johnston et al., 2007 [28]	San Francisco, California (USA)	16 Primary Care clinics	California and ABCD vs. ABCD2	Cohort	962	March 1998–February 1999	Not reported	53
Johnston et al., 2007 [28]	Oxfordshire, UK	9 family practices	California and ABCD vs. ABCD2	Cohort	545	April 2002–March 2005	Not reported	55
Johnston et al., 2007 [28]	Oxfordshire, UK	Hospital-based TIA clinic	California and ABCD vs. ABCD2	Cohort	315	April 2002–March 2005	Not reported	54

**Table 3:** Summarized results from the mixed effects meta-analysis model at the optimal cut-off threshold.

Score	Cut-Off Value	Sensitivity (95% Cr)	Specificity (95% Cr)
ABCD	≥4	0.64 (0.51, 0.74)	0.62 (0.52, 0.71)
ABCD2	≥4	0.59 (0.46, 0.71)	0.62 (0.53, 0.71)
ABCD3-I	≥7	0.53 (0.31, 0.74)	0.68 (0.58, 0.77)
California Score	≥2	0.63 (0.49, 0.74)	0.59 (0.51, 0.67)
ESRS	≥3	0.62 (0.41, 0.79)	0.72 (0.64, 0.79)
SPI-II	≥6	0.48 (0.28, 0.68)	0.64 (0.56, 0.72)

**Table 4:** Summarized results from the network meta-analysis of the diagnostic odds ratio of each score.

Score	Diagnostic Odds Ratio	Between-Study Heterogeneity SD Sensitivity	Between-Study Heterogeneity SD Specificity
ABCD	3.08	0.16 (0.00, 0.86)	0.56 (0.14, 0.97)
ABCD2	2.30	0.70 (0.21, 0.98)	0.60 (0.17, 0.97)
ABCD3-I	4.44	0.42 (0.03, 0.96)	0.49 (0.10, 0.96)
California	2.87	0.40 (0.05, 0.96)	0.43 (0.10, 0.95)
ESRS	3.47	0.47 (0.01, 0.99)	0.50 (0.03, 0.97)
SPI-II	1.69	0.53 (0.04, 0.97)	0.52 (0.03, 0.97

## Data Availability

Data can be made available from the corresponding author on request.

## Supplementary Materials

The following supporting information can be downloaded at https://www.mdpi.com/article/10.3390/jcm14176268/s1. Table S1: Demographics and vascular risk factors in participants of the studies included in the meta-analysis.

## Abbreviations

The following abbreviations are used in this manuscript:

TIA	Transient Ischemic Attack
CVA	Cerebrovascular Accident
ED	Emergency Department
MRI	Magnetic Resonance Imaging
DWI	Diffusion-Weighted Imaging
AUC	Area Under the Curve
SROC	Summary Receiver Operating Characteristic
NMA	Network Meta-Analysis
CrI	Credible Interval
ABCD	Age, Blood pressure, Clinical features, Duration of symptoms
ABCD2	Age, Blood pressure, Clinical features, Duration of symptoms, Diabetes
ABCD3	ABCD2 plus Dual TIA events (≥2 in 7 days), and imaging
ABCD3-I	ABCD3 plus Imaging (carotid stenosis and DWI positivity)
ESRS	Essen Stroke Risk Score
SPI-II	Stroke Prognosis Instrument II
QUIPS	Quality in Prognostic Studies
CHARMS	Checklist for Critical Appraisal and Data Extraction for Systematic Reviews of Prediction Modelling Studies
TP	True Positive
FP	False Positive
TN	True Negative
FN	False Negative
PRISMA-NMA	Preferred Reporting Items for Systematic Reviews and Meta-Analyses for Network Meta-Analyses
PROSPERO	International Prospective Register of Systematic Reviews
