# Supplementary material for: Performance of Stratification Scores on the Risk of Stroke After a Transient Ischemic Attack: A Systematic Review and Network Meta-Analysis
Source: J Clin Med. 2025 Sep 5;14(17):6268. doi: 10.3390/jcm14176268 (PMC12429237; doi:10.3390/jcm14176268)
Supplement: Supplementary file 1 [file jcm-14-06268-s001.zip › jcm-3759584-supplementary.pdf]

Supplementary Table S1. Demographics and vascular risk factors in participants of the studies included in the meta-analysis.

Study	Location	Participants	Mean Age	% Female	Vascular Risk Factors				
					HT %	DM %	Prior Stroke %	Afib %	Smoking
Dai et al, 2015 [21]	Nanjing, China	658	62	26.7	64	18.7	10.5	17.9	34.7
De Marchis et al, 2014 [22]	Switzerland & Germany	302	69	37.1	68.9	11.9	8.3	11.3	20.9
Kiyohara et al, 2014 [23]	Fukoka, Japan	693	69	37.8	75.8	23.5	20.3	18.3	54.3
Knoflach et al, 2016 [24]	Austria	2457	71.9	34.3	78.5	23	Not Reported	19.3	19.5
Song et al, 2013 [25]	Zhengzhou, China	239	57.4	40.2	53.1	14.2	Not Reported	1.7	30.5
Ildstad et al, 2021 [4]	Norway	305	68	40	45.9	10.8	26.9	9.5	55.7
Engelter et al, 2011 [26]	Switzerland	248	70	40	71	17	Not Reported	15	21
Liu et al, 2013 [27]	Shikiazhuang, China	167	61.1	28.7	76.6	35.9	65.9		41.9
Johnston et al, 2007 [28]	San Francisco, California (USA)	1707	Not Reported	53	58	19	23	9	14
Johnston et al, 2007 [28]	Oxfordshire, UK	209	Not Reported	46	38	4	0	14	29
Johnston et al, 2007 [28]	San Francisco, California (USA)	1069	Not Reported	52	57	19	Not Reported	7	Not Reported
Johnston et al, 2007 [28]	San Francisco, California (USA)	962	Not Reported	53	54	18	12	6	18
Johnston et al, 2007 [28]	Oxfordshire, UK	547	Not Reported	55	43	9	12	11	12
Johnston et al, 2007 [28]	Oxfordshire, UK	315	Not Reported	54	50	10	8	5	21
